# Lovastatin Enhances Cytotoxicity of Temozolomide via Impairing Autophagic Flux in Glioblastoma Cells

**DOI:** 10.1155/2019/2710693

**Published:** 2019-09-23

**Authors:** Zhiyuan Zhu, Pingde Zhang, Ning Li, Karrie Mei Yee Kiang, Stephen Yin Cheng, Vincent Kam-Wai Wong, Gilberto Ka-Kit Leung

**Affiliations:** ^1^Division of Neurosurgery, Department of Surgery, Li Ka Shing Faculty of Medicine, The University of Hong Kong, Hong Kong, China; ^2^State Key Laboratory of Quality Research in Chinese Medicines, Macau University of Science and Technology, Macau, China

## Abstract

Drug resistance to temozolomide (TMZ) contributes to the majority of tumor recurrence and treatment failure in patients with glioblastoma multiforme (GBM). Autophagy has been reported to play a role in chemoresistance in various types of cancer, including GBM. The anticancer effect of statins is arousing great research interests and has been demonstrated to modulate autophagic function. In this study, we investigated the combinational effects of lovastatin and TMZ on treating U87 and U251 GBM cell lines. Cytotoxicity was measured by MTT and colony formation assays; apoptosis was measured by flow cytometry; the cellular autophagic function was detected by the EGFP-mRFP-LC3 reporter and western blot assay. The results showed that lovastatin might enhance the cytotoxicity of TMZ, increase the TMZ-induced cellular apoptosis, and impair the autophagic flux in GBM cells. Lovastatin triggered autophagy initiation possibly by inhibiting the Akt/mTOR signaling pathway. Moreover, lovastatin might impair the autophagosome-lysosome fusion machinery by suppressing LAMP2 and dynein. These results suggested that lovastatin could enhance the chemotherapy efficacy of TMZ in treating GBM cells. The mechanism may be associated with impaired autophagic flux and thereby the enhancement of cellular apoptosis. Combining TMZ with lovastatin could be a promising strategy for GBM treatment.

## 1. Introduction

Glioblastoma multiforme (GBM) is the most common and malignant primary brain tumor in adults. The annual incidence rate of GBM is 3.19/100,000, accounting for more than 60% of all gliomas [1, 2]. The median survival is only around 14 months after diagnosis despite standard treatment regimen consisting of surgical resection, radiation, and chemotherapy [3]. Tumor progression and recurrences are almost inevitable in patients with GBM, rendering it one of the most devastating cancers in human.

Temozolomide (TMZ) is an oral DNA alkylating agent that is used in combination with radiotherapy for treating patients with newly diagnosed GBM [4]. TMZ induces DNA methylation at purine bases like O6-guanine, N7-guanine, or N3-adenine. Methylated nucleobase leads to mismatched base pairs and persistent DNA double-strand breaks, which consequentially result in cell cycle arrest, autophagy, and cell apoptosis [5]. However, TMZ-induced autophagy may also act as a survival mechanism that contributes to chemotherapy resistance in GBM [6].

Autophagy is a catabolic process that supplies cells with energy and raw materials for biosynthesis by recycling degraded proteins and damaged organelles. It can be triggered when cells are under stress like nutrient deprivation, chemotherapy drugs, or metabolic stress. Inhibition of autophagy either at the initiation stage or the late autolysosome fusion stage could enhance TMZ-induced apoptosis, suggesting that the suppression of autophagy might improve the outcome of TMZ-based chemotherapy [7, 8]. A complicating factor is that, in some other contexts, autophagy might cause cellular apoptosis and cell death, rendering it necessary to examine the precise effect of individual autophagy modulators in each tumor type [9].

Statins are among the widest used drugs in patients with cardiovascular and coronary heart diseases. Their canonical role is lowering the serum cholesterol level by inhibiting 3-hydroxy-3-methylglutaryl coenzyme A (HMG-CoA) reductase. Recently, statins have emerged as potential anticancer agents with antiproliferative, proapoptotic, anti-invasive, and radio-sensitizing functions in various types of cancer [10, 11]. Parikh et al. reported that statins regulated autophagic function and caused autophagy-associated cell death in prostate cancer cells [12]. In glioma, statins were also reported to inhibit cell proliferation and migration and increase apoptosis [13, 14]. Moreover, lovastatin was reported to sensitize GBM cells to tumor necrosis factor- (TNF-) related apoptosis-inducing ligand (TRAIL) induced apoptosis [15]. Atorvastatin enhanced TMZ's efficacy in GBM via prenylation-dependent inhibition of Ras signaling [16]. Whether and how lovastatin may act in combination with TMZ in treating GBM has not been explored. This study aimed to investigate the effects of concomitant use of lovastatin with TMZ on GBM cells *in vitro*. The hypothesis was that lovastatin would enhance the cytotoxicity of TMZ in GBM cells through the inhibition of autophagy function.

## 2. Materials and Methods

### 2.1. Cell Culture and Drug Treatment

Human GBM cell lines U87 and U251 (American Type Culture Collection, Manassas, VA, USA) were maintained in MEM*α* medium (Gibco, Invitrogen, Grand Island, NY, USA) with 10% fetal bovine serum, 100 IU/ml penicillin, and 100 *μ*g/ml streptomycin (Gibco). U87 cells with EGFP-mRFP-LC3 were obtained from Professor Vincent Kam-Wai Wong from Macau University of Science and Technology. Cells were treated with 5 *μ*M lovastatin and/or 500 *μ*M TMZ for 72 hrs in the experiments besides the MTT assay. All cells were cultured in a 5% CO_2_ humidity incubator at 37°C.

The real-time PCR-based mycoplasma screening test was performed monthly by the Faculty Core Facility of our institution to ensure that the cultured cells were mycoplasma free. Both cell lines were authenticated by short tandem repeat (STR) analysis (performed by Genetica cell line testing, LabCorp, NC, USA).

### 2.2. Reagents and Antibodies

Temozolomide (Schering-Plough, NJ, USA) was dissolved in DMSO to make a stock of 10 mM. Lovastatin was obtained from Sigma (St. Louis, MO, USA) and dissolved in DMSO at a concentration of 10 mM. Thiazolyl blue tetrazolium bromide (MTT), DMSO, and bafilomycin-A1 were obtained from Sigma. The primary antibodies were purchased from Cell Signaling Technology (CST, Beverly, MA, USA) and Santa Cruz Biotechnology (SC, Santa Cruz Biotechnology, Inc., Dallas, Texas, USA) and diluted in 5% bovine serum albumin (BSA) according to the manufacturer's instructions: SQSTM1/p62 (CST #5114, 1 : 1000), LC3A/B (CST #12741, 1 : 1000), cleaved caspase-3 (CST #9661, 1 : 1000), cleaved PARP (CST #5625, 1 : 1000), Bcl-2 (CST #4223, 1 : 1000), Bim (CST #2933, 1 : 1000), Akt (CST #4691, 1 : 1000), p-AKT (CST #4060, 1 : 1000), mTOR (CST #2983, 1 : 1000), GAPDH (CST #5174, 1 : 1000). LAMP1 (SC-20011, 1 : 1000), LAMP2 (SC-18822, 1 : 1000), and dynein (SC-514579, 1 : 1000). The secondary antibodies included anti-rabbit IgG, HRP-linked antibody (CST #7074, 1 : 5000), and mouse IgG HRP linked whole Ab (GE Healthcare, NA931, 1 : 5000).

### 2.3. Cell Viability Assay

Cytotoxicity of lovastatin and/or TMZ treatment was measured by the MTT assay. Briefly, 4000 cells/well were seeded onto 96-well plates for 24 hrs and then treated with different dosages of lovastatin, TMZ, or both. After incubation for 72 hrs, 10 *μ*l MTT reagent (5 mg/ml) was added into each well and incubated at 37°C for 3 hrs. The supernatants were gently aspirated, and the formazan crystals were dissolved in DMSO for 15 min. Absorbance at 595 nm was measured by the Thermo Varioskan flash reader. The half-maximum inhibitory concentration (IC_50_ values) was derived from the best-fit curve using nonlinear regression. Cells in each group were plated in triplicate; three independent experiments were performed.

### 2.4. Colony Formation Assay

Cells were seeded into 6-well plates at a density of 500 cells/well, and they were treated with lovastatin, TMZ, or both for 72 hrs before transferring to fresh medium. Cells were incubated for a total of two weeks. Colonies were then fixed with 75% ethanol, stained with crystal violet (0.5% w/v), and counted under the microscope. A colony is defined to be consisting of at least 50 cells.

### 2.5. Immunoblotting

Cells were harvested and total protein extracted with RIPA lysis buffer (Cell Signaling Technology) containing 10% protease inhibitor (Roche Diagnostics, Indianapolis, IN, USA). Cell lysates were separated in SDS-PAGE and then transferred to PVDF membrane. The membrane was blocked by 5% non-fat milk and then incubated with the appropriate primary antibody at 4°C overnight. After washing with TBST (tris-buffered saline with 0.1% Tween 20) thrice, the membrane was incubated with HRP-linked secondary antibodies at room temperature for 1 hr. Antibody binding was detected by enhanced chemiluminescent HRP substrate (ECL) detection kit (Millipore Corporation, MA, USA). Bands were exposed to X-ray film. Quantification of western blot results was analyzed by Image J (National Institutes of Health, Maryland, US). The intensity of western blot bands was measured and normalized to the value of GAPDH.

### 2.6. Calculation of Autophagosome and Autolysosome Numbers

U87 cells with EGFP-mRFP-LC3 expression were seeded onto coverslips in 48-well plates. After treatment, cells were washed by PBS (phosphate-buffered saline) twice and then fixed by 70% ethanol for 30 min. The coverslips were mounted by Fluoromount-G, with DAPI (Invitrogen by Thermo Fisher Scientific, MA, United States) and observed under a confocal microscope. The method of calculating the autophagosome and autolysosome numbers was described [17]. Briefly, the puncta formation assay counts the average number of punctate structures per cell by using the confocal fluorescence microscope. In each group, the average number of the visible punctate structures per cell was calculated by counting three randomly selected view fields. The autophagosomes were labelled as yellow puncta (GFP-positive/RFP-positive), and the matured autolysosomes were red (GFP-negative/RFP-positive). The percentage of red puncta was calculated as follows: red/(red + yellow).

### 2.7. Flow Cytometry Analysis of Apoptosis

Cell apoptosis was measured by Annexin V/propidium iodide (PI) double staining according to the manufacturer's instructions (Roche Diagnostics, Indianapolis, IN, USA). Briefly, cells were collected and washed with cold PBS and then resuspended in 100 *μ*l binding buffer containing 2 *μ*l of Annexin V and 2 *μ*l of PI. Samples were incubated at room temperature and protected from light for 15 min. Another 500 *μ*l binding buffer was added into each sample tube after incubation, and cells were analyzed by FACS (Beckman Coulter, CA). The signal of 10,000 cells was recorded and analyzed. The percentage of apoptotic cells included both early apoptotic cells (Annexin V+/PI−) and late apoptotic/necrotic cells (Annexin V+/PI+).

### 2.8. Statistical Analysis

All statistical analyses were performed using GraphPad Prism 7.0 (GraphPad Software Inc.). One-way ANOVA with Tukey's multiple comparisons test was used to evaluate differences among groups. A *p* value less than 0.05 was considered statistically significant. Results were stated as mean ± standard deviation (SD).

## 3. Results

### 3.1. Lovastatin Enhanced Cytotoxicity of TMZ in GBM Cells

**Figure 1:**
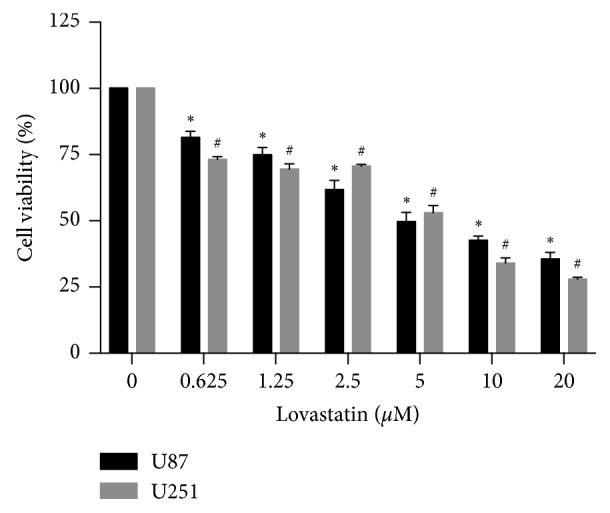
MTT results showed the toxicity of lovastatin in GBM cells. U87 and U251 cells were treated with different concentrations of lovastatin for 72 hrs. Cell viability was measured by the MTT assay. Dosage at 5 *μ*M represented the IC_50_ value of lovastatin in both cells lines. Therefore, it was chosen as the working concentration in further experiments; *N* = 3. ^*∗*^*p* < 0.05 compared with control in U87 cells; ^#^*p* < 0.05 compared with control in U251 cells.

**Figure 2:**
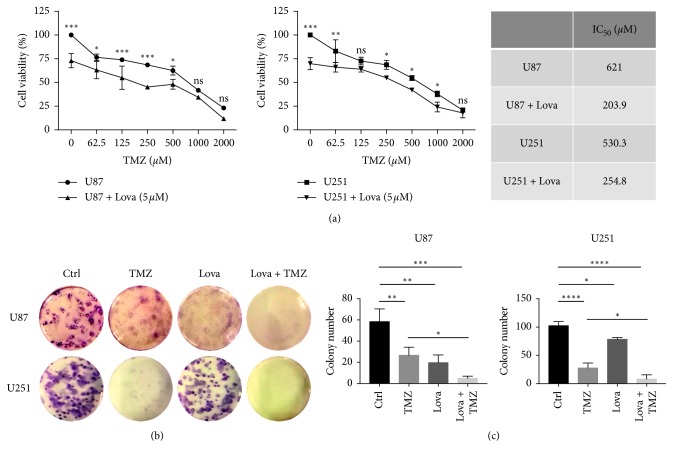
Lovastatin enhanced the cytotoxicity of TMZ in GBM cells. (a) U87 and U251 cells were treated with different concentrations of TMZ with or without lovastatin (5 *μ*M) for 72 hrs. The IC_50_ value of TMZ was measured by the MTT assay. Cells treated with TMZ + lovastatin were compared with TMZ alone at each TMZ concentration; *N* = 3. (b) Colony formation assay. Cells (500 cells/well) were treated with TMZ (200 *μ*M), lovastatin (2.5 *μ*M), or combination for 3 days and then cultivated with fresh medium. The cells were cultivated for 2 weeks in total. Colonies were counted under a light microscope. (c). Quantification of colony formation results; *N* = 3. ^*∗*^*p* < 0.05; ^*∗∗*^*p* < 0.01; ^*∗∗∗*^*p* < 0.001; ^*∗∗∗∗*^*p* < 0.0001; ns = no significance.

Lovastatin showed dose-dependent cytotoxicity on GBM cells. The IC_50_ values of lovastatin on U87 and U251 cells were around 5 *μ*M (6.1 *μ*M and 5.1 *μ*M, respectively) (Figure 1). Therefore, 5 *μ*M of lovastatin was chosen for use in the subsequent experiments. We then investigated the cytotoxicity of combinatorial treatments with lovastatin and TMZ. The results showed that cotreatment significantly decreased the IC_50_ values when compared to TMZ alone in both cell lines (203.9 *μ*M vs. 621 *μ*M in U87 cells and 254 *μ*M vs. 530.3 *μ*M in U251 cells) (Figure 2(a)). The colony formation assay showed that lovastatin enhanced the chronic cytotoxicity of TMZ. As shown in Figures 2(b) and 2(c), TMZ alone inhibited colony formation in both U87 and U251 cells; the addition of lovastatin further reduced the colony number by 33.7% and 45%, respectively. These data were consistent with the previous studies that showed atorvastatin dose-dependently suppressed growth and survival in GBM cells and enhanced TMZ's chemotherapy efficacy [13, 16]. Together, our results suggested that lovastatin, similar to atorvastatin, could enhance the chemotherapy efficacy of TMZ in GBM cells in both short and long terms.

### 3.2. Lovastatin Increased TMZ-Induced Cellular Apoptosis in GBM Cells

**Figure 3:**
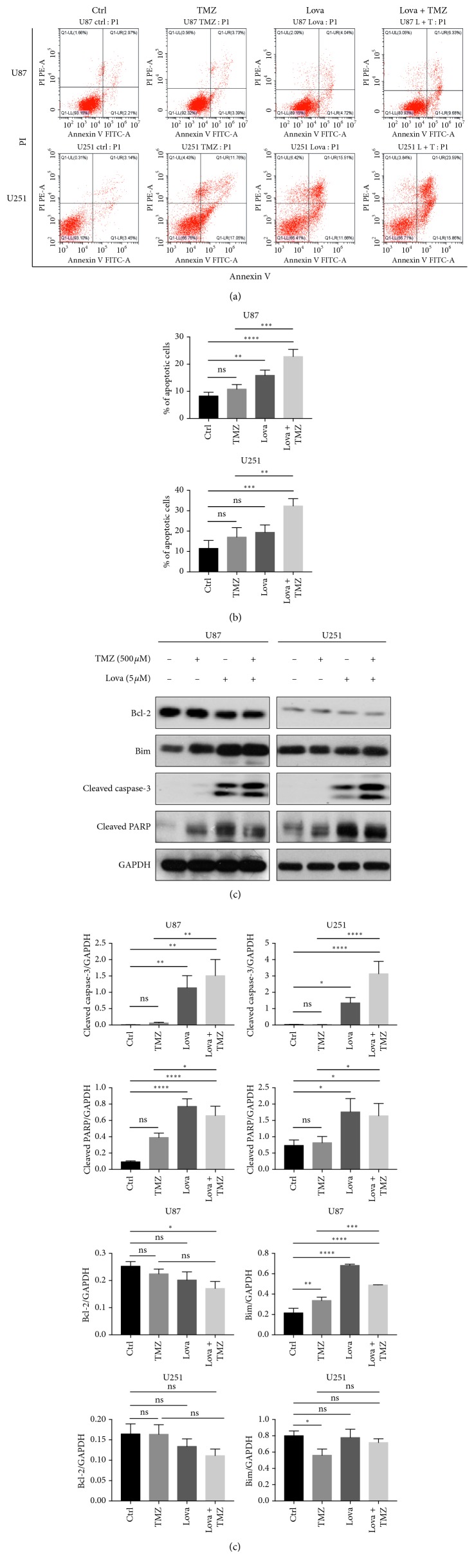
Lovastatin increased TMZ-induced cellular apoptosis in GBM cells. (a) U87 and U251 cells were treated with TMZ (500 *μ*M), lovastatin (5 *μ*M), or combination for 72 hrs; the cellular apoptotic level was detected by Annexin V/PI staining. (b) Quantification of flow cytometry-measured cell apoptotic levels; *N* = 3. (c) Apoptotic markers were detected by the western blot assay. GAPDH was used as an internal loading control. (d) Quantification of western blot results of apoptotic markers; *N* = 3. ^*∗*^*p* < 0.05; ^*∗∗*^*p* < 0.01; ^*∗∗∗*^*p* < 0.001; ^*∗∗∗∗*^*p* < 0.0001; ns = no significance.

TMZ treatment caused DNA double-strand break, which could trigger DNA repair response and cell apoptosis [18]. Statins were reported to induce apoptosis by increasing DNA fragmentation, thereby activating proapoptotic gene *Bax* and inhibiting *Bcl-2* [19, 20]. Additionally, statins induced the hematopoietic tumor cell apoptosis through enhancing the expression of proapoptotic gene *Bim* [21]. We then asked whether or not lovastatin could increase the GBM cells apoptotic level caused by TMZ. Flow cytometry analysis showed that, when compared with control groups, TMZ-alone treatment did not cause a statistically significant rise in apoptosis in U87 or U251 cells. Lovastatin alone significantly increased the cellular apoptosis in U87 cells, while a similar trend was observed in U251 cells. Importantly, when compared to TMZ alone, cotreatment strikingly increased the percentages of the apoptotic cell population by 11.96% and 15.26%, respectively (Figures 3(a) and 3(b)). On immunoblotting assays, when compared to TMZ alone, our cotreatment significantly increased the cleavage of caspase-3 and PARP in both U87 and U251 cells, suggesting increased cellular apoptotic levels (Figures 3(c) and 3(d)). When compared with the control group, lovastatin alone increased the level of proapoptotic gene *Bim* in U87 but not in U251 cells. The level of antiapoptotic gene Bcl-2 was not significantly different between lovastatin and control groups. Altogether, these results suggested that while lovastatin alone might cause apoptosis in GBM cells, the combinatorial treatment could result in a further and significant increase.

### 3.3. Lovastatin Triggered Autophagy Initiation but Blocked Autophagic Flux in GBM Cells

Statins were reported to trigger autophagy in various types of cancer, but their regulatory role on autophagic flux—the actual dynamic process of autophagy—has not been thoroughly examined. Lipid-bound LC3-II is the marker of autophagosomes which is converted from the soluble LC3-I; p62/SQSTM1 is one of the substrates that degraded in autolysosomes. Typically, the induction of autophagy was indicated by increased turnover of LC3-I to LC3-II; and the degradation of substrates in autolysosomes (e.g., p62) could indicate the fusion of autophagosomes and lysosomes [22].

**Figure 4:**
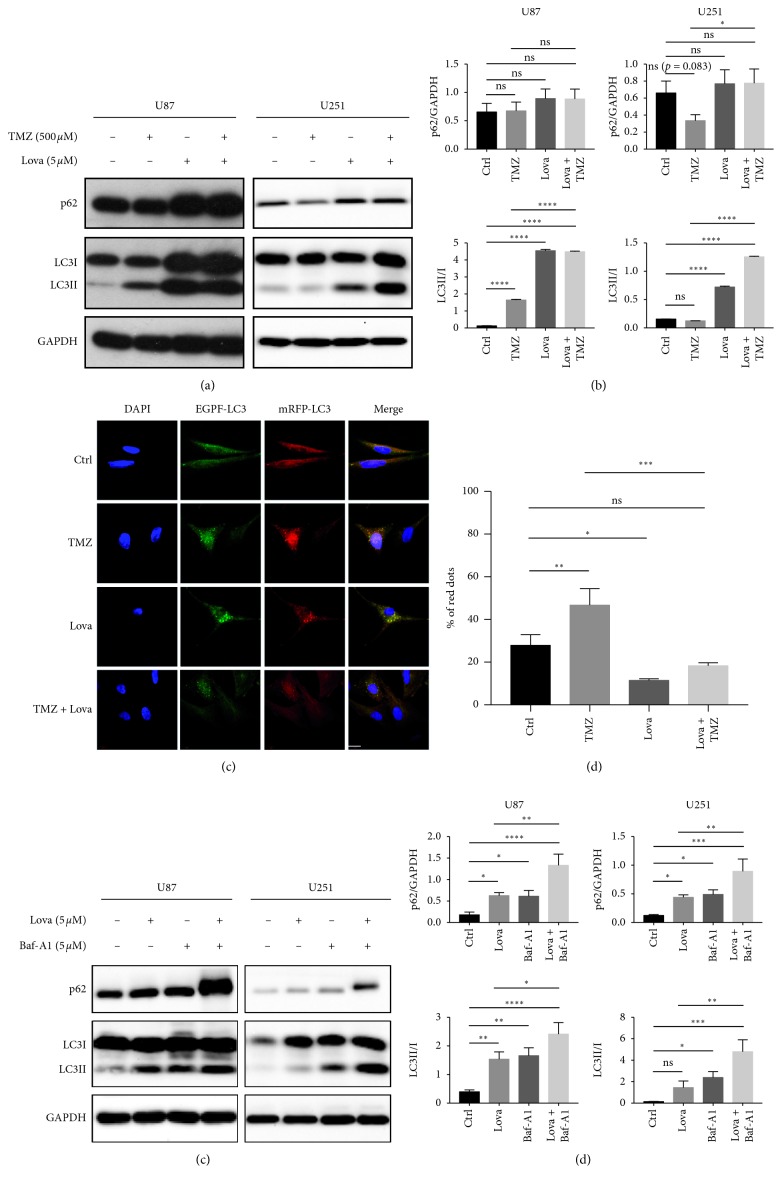
Lovastatin triggered autophagy but blocked autophagic flux in GBM cells. (a) U87 and U251 cells were treated with TMZ (500 *μ*M), lovastatin (5 *μ*M), or combination for 72 hrs, and autophagic markers (LC3I/II and p62) were detected by western blot. (b) Quantification of western blot results in (a); *N* = 3. (c) U87 cells expressing EGFP-mRFP-LC3 were treated with TMZ (500 *μ*M), lovastatin (5 *μ*M), or combination for 72 hrs. Fluorescence images were taken by using a confocal microscope (scale bar = 20 *μ*m). The average number of red and yellow puncta per cell was calculated from three random-picked view fields. (d) Quantification of results in (c); *N* = 3. (e) U87 and U251 cells were treated with lovastatin (5 *μ*M), bafilomycin-A1 (5 *μ*M), or combination for 72 hrs, and autophagic markers were detected by western blot. (f) Quantification of western blot results in (e); *N* = 3. ^*∗*^*p* < 0.05; ^*∗∗*^*p* < 0.01; ^*∗∗∗*^*p* < 0.001; ^*∗∗∗∗*^*p* < 0.0001; ns = no significance.

The results showed that lovastatin-alone treatment significantly increased the LC3 turnover (LC3-I to LC3-II) when compared to control in U87 and U251 cells, while no significant difference in the p62 level was observed between lovastatin and control groups. Concomitant treatment increased both LC3II and p62 levels in U251 cells when compared with the TMZ-alone group. U87 cells showed a similar trend, although the difference in the p62 level was not statistically significant (Figures 4(a) and 4(b)). When compared with the control groups, TMZ treatment increased the LC3-II turnover in U87 cells but not in U251 cells, while the p62 level showed a decreasing trend in U251 cells (no statistical significance) and no difference in U87 cells (Figures 4(a) and 4(b)). These results suggested that TMZ-alone and lovastatin-alone treatment would increase the LC3 turnover, indicating the induction of autophagy. However, lovastatin-induced autophagy might be suppressed at the substrate-degradation level and not fully functional with or without TMZ.

We then used U87 cells with the EGFP-mRFP-LC3 tandem reporter (tfLC3) to examine the autophagic flux. Typically, the neutral pH value within autophagosomes allows the manifestations of both GFP and RFP signals, which appear as yellow puncta in the merged phase. However, the GFP fluorescence is quenched under the autolysosome's acidic condition, leaving only RFP's red signal. Thus, the percentage of red puncta would indicate the proportion of matured autolysosomes [23]. We found that lovastatin-alone treatment decreased the proportion of single red puncta when compared to the control group. Our cotreatment significantly reduced the percentage of single red puncta when compared to TMZ alone (Figures 4(c) and 4(d)). The tfLC3 tracking data showed a suppression of autolysosome maturation caused by lovastatin treatment, suggesting that lovastatin might impair the autophagic flux by suppressing autolysosome fusion.

Bafilomycin A1 is a lysosomal protease inhibitor widely used to cancel lysosomal acidification by specifically inhibiting the vacuolar type ATPase [23]. Bafilomycin A1 treatment blocks the degradation of lysosome substrates and leads to the accumulation of both LC3-II and p62. Thus, it is commonly used to investigate the autophagic flux. We firstly showed that bafilomycin A1 treatment in GBM cells increased the levels of both LC3-II and p62 when compared to control; these data are compatible with the antilysosome function of bafilomycin A1. Lovastatin treatment showed similar effects, supporting its role in suppressing lysosome. Strikingly, the combination of lovastatin and bafilomycin A1 significantly increased LC3-II as well as p62 levels when compared with either bafilomycin A1-alone or lovastatin-alone treatment (Figures 4(e) and 4(f)). These data suggested that lovastatin showed comparable lysosomal inhibition effects with bafilomycin A1 and the concomitant treatment further blocked the degradation of LC3-II and p62 in lysosomes.

### 3.4. Lovastatin-Impaired Autophagic Flux Might Be Mediated via the Inhibition of the Akt/mTOR Signaling Pathway and Suppression of Autophagosome-Lysosome Fusion Machinery

**Figure 5:**
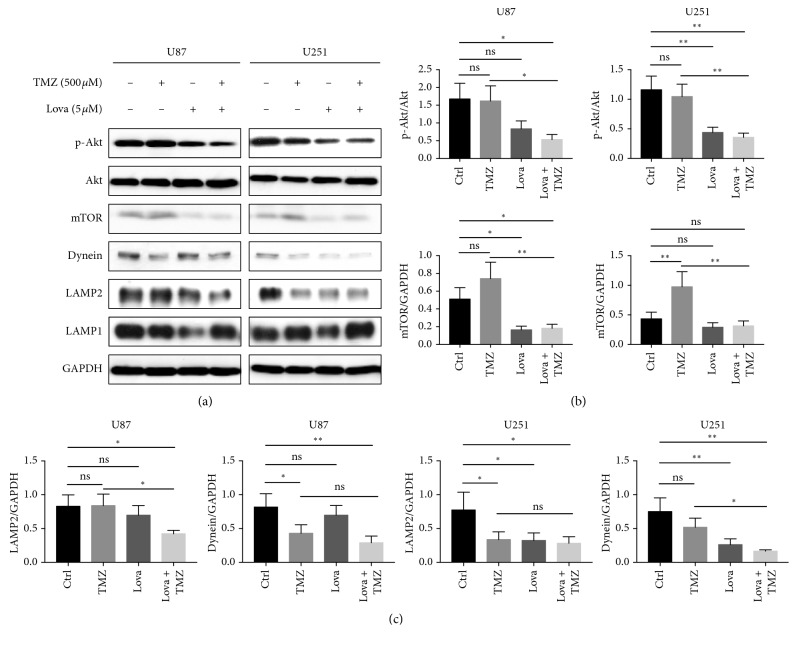
Lovastatin triggered the autophagy initiation pathway and impaired autophagosome-lysosome fusion mechanism. U87 and U251 cells were treated with TMZ (500 *μ*M), lovastatin (5 *μ*M), or combination for 72 hrs. (a) The western blot assay was used to detect the key markers in the autophagic initiation pathway and autolysosome maturation. (b) Quantification of western blot results of the Akt/mTOR pathway; *N* = 3. (c) Quantification of western blot results of autolysosome fusion markers; *N* = 3. ^*∗*^*p* < 0.05; ^*∗∗*^*p* < 0.01; ^*∗∗∗*^*p* < 0.001; ^*∗∗∗∗*^*p* < 0.0001; ns = no significance.

We then investigated the potential molecular mechanisms underlying the regulatory role of lovastatin on autophagy. The western blot assay showed that, when compared to the control group, lovastatin-alone treatment led to a decrease of p-Akt in U251 cells and a decline of mTOR in U87 cells. Cotreatment with lovastatin and TMZ significantly decreased p-Akt and mTOR expressions in both U87 and U251 when compared to TMZ alone (Figures 5(a) and 5(b)). These data indicated that lovastatin treatment might active the canonical autophagy induction Akt/mTOR pathway. LAMP1/2 and dynein are important mediators required for the process of lysosome-autophagosome fusion [24, 25]. We found that, when compared to the control group, lovastatin decreased the level of LAMP2 and dynein in U251 cell and a similar trend was observed in U87 cells (no statistical significance). When compared to TMZ alone, cotreatment groups had a lower level of LAMP2 in U87 cells and lower dynein level in U251 cells (Figures 5(a) and 5(c)). Thus, lovastatin treatment suppressed the LAMP2 and dynein levels, which might contribute to the block of autolysosome fusion.

## 4. Discussion

In this study, we found that concomitant treatment with lovastatin could enhance the efficacy of TMZ in GBM cells in vitro by impairing cellular autophagic flux. The cotreatment inhibited GBM cell viability and increased cellular apoptosis. Lovastatin impaired the autophagic flux by inducing autophagy initiation but blocking autolysosome maturation.

Statins have been widely studied as anticancer agents. Epidemiological studies reported that statin use might inversely correlate with the risk of various types of cancer [26] including that of glioma [27–29]. Furthermore, statin use reduced cancer-related mortality and improved the survival of GBM patients [30, 31]. However, several retrospective studies had inconsistent conclusions that statin use was not associated with the risk of glioma or with the survival of patients with glioma [32–34]. Thus, further experimental and epidemiological studies are needed to support the rationale for prospective studies on the possible anticancer role of statins within the concept of drug repurposing.

Laboratory evidence suggested that statins exhibited cancer therapy potential and radiosensitizing effects when used in conjunction with radiotherapy for the treatment of prostate cancer [35] and squamous cell carcinoma [36]. Combination of statins with thiazolidinediones also showed a synergistic cytotoxic effect against glioblastoma cells both in vitro and in vivo [37, 38]. We, therefore, surmised that lovastatin would enhance the efficacy of TMZ in GBM cells. The clinical relevance and significance of this research question lie in the fact that TMZ is now a standard treatment for GBM. In line with the previous studies, our data showed that lovastatin caused GBM cell death in a dose-dependent manner and enhanced the cytotoxicity of TMZ in both short and long terms (Figures 1 and 2). Moreover, concomitant lovastatin acted synergistically with TMZ on inducing cell apoptosis (Figure 3), suggestive of lovastatin's potential role as a chemoenhancer in the treatment of GBM. Regarding the role of TMZ in regulating autophagy in GBM cells, our data showed that TMZ increased the LC3 turnover in U87 cells and decreased the p62 level in U251 cells. These results were partially compatible with the previous reports in which the autophagy induction had been found to be a putative mechanism of TMZ action in GBM cells [6]. Other investigators also reported that LC3 was recruited in TMZ-induced autophagy in U373 and U87 GBM cell lines [39, 40]. However, how TMZ may affect autophagic flux dynamic process remains unclear. Whether TMZ-induced autophagy would lead to either cancer cell survival or cell death is likely to be dependent on the specific cellular context.

The mechanisms underlying statins' antitumor effects remain unclear. It has been reported that statins might cause cell apoptosis and increase the autophagy in cancer cells. In C6 glioma cells, several types of statins consistently induced cellular apoptosis and decreased the level of phosphorylated ERK1/2 and Akt [41]. Atorvastatin treatment dose-dependently inhibited growth and survival of multiple GBM cells, and it significantly enhanced TMZ's efficacy both in vitro and in vivo [16]. Moreover, atorvastatin promoted the occurrence of autophagosome markers and increased the apoptosis in A172 glioma cells [13]. Autophagy is widely considered as a cell protective mechanism in the face of cell stress. However, the impairment of the dynamic process of autophagy flux could also lead to cell death [42]. Whether statin-induced autophagy is fully functional or statins might actually induce cell death in this regard has not yet been determined. We therefore investigated the effects of lovastatin on autophagy function in GBM cells. Typically, functional autophagy consists of two steps—the formation of autophagosomes and their fusion with lysosomes—in a dynamic process of “autophagic flux”. The induction of autophagy is indicated by an increased turnover of LC3-I to LC3-II; any rise in the degradation of autolysosomes substrates (e.g., p62) would indicate autophagosome-lysosome fusion [22, 43]. In line with the previous reports, we found that lovastatin-alone treatment increased the cellular apoptosis in glioma cells (Figure 3). However, while lovastatin remarkably increased the autophagosome marker LC3II when compared with the control groups, it did not cause any difference in substrate degradation (i.e., SQSTM1/p62 level) (Figures 4(a) and 4(b)). Our findings suggested an increased LC3 turnover and autophagy induction but not p62 degradation, nondegraded p62 indicated, leading us to surmise that lovastatin might play a more complex role in autophagic flux than acting solely as an autophagy initiator.

To dissect this dynamic process, we used the tandem tfLC3 to track the autophagic flux by separately detecting autophagosomes and autolysosomes. The reduced proportion of matured autolysosomes (red puncta) attributed to lovastatin treatment, suggesting that lovastatin might impair the autophagic function by inhibiting the maturation of autolysosomes (Figures 4(c) and 4(d)). Lovastatin also showed lysosomal inhibition effects (increased LC3-II and p62 levels) similar to bafilomycin A1, a lysosomal inhibitor widely used in the autophagy research field. Their concomitant treatment further blocked the degradation function of lysosomes (Figures 4(e) and 4(f)). Taken together, lovastatin would, on the one hand, induce the autophagic initiation through enhanced autophagosome formation in GBM cells; while on the other hand, it inhibited the fusion of autophagosomes and lysosomes, resulting in impaired autophagic function.

**Figure 6:**
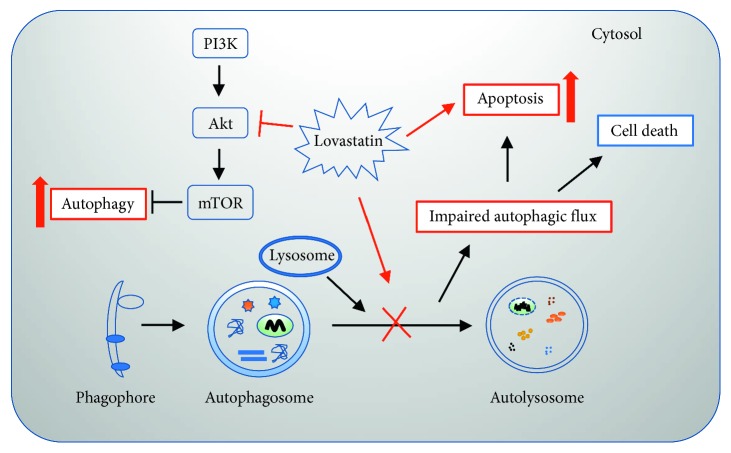
Schematic diagram of the hypothetical mechanism for lovastatin-induced cytotoxicity in GBM cells. Lovastatin treatment initiated autophagy, which might be mediated via suppressing the Akt/mTOR pathway. The fusion of autophagosome and lysosome was blocked by lovastatin. The impaired autophagic flux led to cellular apoptosis and cell death in GBM cells.

To date, the mechanism of statins regulating the autophagic flux is incompletely understood. It has been reported that stains might activate autophagy via the AMPK-mTOR signaling pathway in astrocytes [44] and enhance autophagy in coronary arterial myocytes through inhibition of the Rac1-mTOR pathway [45]. Our results showed that, while lovastatin might trigger autophagy possibly by inhibiting the Akt-mTOR pathway (Figures 5(a) and 5(b)), it may inhibit the autolysosome fusion machinery (and therefore autophagic flux) by inhibiting two critical mediators, LAMP2 and dynein (Figures 5(a) and 5(c)). As illustrated in Figure 6, lovastatin triggered the autophagic initiation pathway but inhibited the autophagosome-lysosome fusion machinery; the subsequent impairment of autophagic flux may lead to cellular apoptosis and cell death.

Intrinsic and acquired resistance to TMZ is the major obstacle in GBM treatment, mainly due to resistance against apoptotic stimuli. Autophagy is generally regarded as a cell-protective process that facilitates chemotherapy resistance, but excessive or impaired autophagy may also be detrimental to cell survival by inducing cellular apoptosis [46] and cell death [42]. Thus, autophagy modulators have been widely studied in the chemotherapy research field. In this study, we found increased cellular apoptosis and impaired autophagic flux after lovastatin treatment in GBM cells (Figures 3 and 4). However, although the relationship between autophagy and apoptosis has been widely studied, their connections are multifaceted and complex. Whether our observed cellular apoptosis was caused by or incidental to dysfunctional autophagic flux was unclear since the causal relationship between autophagy and apoptosis has yet been adequately demonstrated. A number of studies reported that, under certain circumstances, autophagy could promote death and itself may be a mechanism of cell death (i.e., “autophagic cell death”) [47, 48], whereas others considered this “autophagic cell death” as merely cell death accompanied by autophagosomes [49]. To further study the bidirectional cross-talk and molecular connections between autophagy and apoptosis in GBM, further researches are needed.

Our proof-of-concept study has several limitations. First, in vitro studies using more relevant in vitro models (primary GBM cells and glioma stem cells) and in vivo studies using xenograft animal models are needed to further verify our findings. Second, Akt/mTOR activators should be used in combination with lovastatin to conclude the mechanism of lovastatin-induced autophagy definitively. Third, the effect of lovastatin on radiation therapy has not been investigated. Clinical trials investigating the use of statins in combination with the chemoirradiation regimen in newly diagnosed GBM patients have been rare. To the best of our knowledge, there has only been one registered phase II clinical trial launched in 2014 to evaluate the efficacy and safety of atorvastatin in combination with radiotherapy and TMZ in GBM patients (NCT02029573); no study report has been released although the trial should have been completed in 2016.

In conclusion, we demonstrated that lovastatin enhanced the chemotherapy efficacy of TMZ in GBM cells in vitro; the underlying mechanism may involve the inhibition of autophagic flux and thereby the enhancement of apoptosis. Given the general accessibility, relatively low cost, and good safety profile of statins, further research and clinical trials are needed to clarify the adjunctive role of statins in GBM treatment.

## Data Availability

The data that support the findings of this study are available from the corresponding author upon request.
